# The effect of diabetes on COVID-19 incidence and mortality: Differences between highly-developed-country and high-migratory-pressure-country populations

**DOI:** 10.3389/fpubh.2023.969143

**Published:** 2023-03-08

**Authors:** Marta Ottone, Letizia Bartolini, Laura Bonvicini, Paolo Giorgi Rossi, Reggio Emilia COVID-19 working group, Massimo Costantini

**Affiliations:** Epidemiology Unit, Azienda USL-IRCCS di Reggio Emilia, Reggio Emilia, Italy

**Keywords:** immigrants, SARS-CoV-2 infection, COVID-19 mortality, type-2 diabetes, diabetes register

## Abstract

The objective of this study was to compare the effect of diabetes and pathologies potentially related to diabetes on the risk of infection and death from COVID-19 among people from Highly-Developed-Country (HDC), including Italians, and immigrants from the High-Migratory-Pressure-Countries (HMPC). Among the population with diabetes, whose prevalence is known to be higher among immigrants, we compared the effect of body mass index among HDC and HMPC populations. A population-based cohort study was conducted, using population registries and routinely collected surveillance data. The population was stratified into HDC and HMPC, according to the place of birth; moreover, a focus was set on the South Asiatic population. Analyses restricted to the population with type-2 diabetes were performed. We reported incidence (IRR) and mortality rate ratios (MRR) and hazard ratios (HR) with 95% confidence interval (CI) to estimate the effect of diabetes on SARS-CoV-2 infection and COVID-19 mortality. Overall, IRR of infection and MRR from COVID-19 comparing HMPC with HDC group were 0.84 (95% CI 0.82–0.87) and 0.67 (95% CI 0.46–0.99), respectively. The effect of diabetes on the risk of infection and death from COVID-19 was slightly higher in the HMPC population than in the HDC population (HRs for infection: 1.37 95% CI 1.22–1.53 vs. 1.20 95% CI 1.14–1.25; HRs for mortality: 3.96 95% CI 1.82–8.60 vs. 1.71 95% CI 1.50–1.95, respectively). No substantial difference in the strength of the association was observed between obesity or other comorbidities and SARS-CoV-2 infection. Similarly for COVID-19 mortality, HRs for obesity (HRs: 18.92 95% CI 4.48–79.87 vs. 3.91 95% CI 2.69–5.69) were larger in HMPC than in the HDC population, but differences could be due to chance. Among the population with diabetes, the HMPC group showed similar incidence (IRR: 0.99 95% CI: 0.88–1.12) and mortality (MRR: 0.89 95% CI: 0.49–1.61) to that of HDC individuals. The effect of obesity on incidence was similar in both HDC and HMPC populations (HRs: 1.73 95% CI 1.41–2.11 among HDC vs. 1.41 95% CI 0.63–3.17 among HMPC), although the estimates were very imprecise. Despite a higher prevalence of diabetes and a stronger effect of diabetes on COVID-19 mortality in HMPC than in the HDC population, our cohort did not show an overall excess risk of COVID-19 mortality in immigrants.

## Introduction

Risk factors increasing COVID-19 mortality can act in two main ways: by increasing the probability of infection or by increasing the severity and lethality of the disease following infection. In addition to biological characteristics, a multitude of social and economic factors can influence both the probability of infection and the severity of the disease. Several studies have shown that mortality from COVID-19, as reported by routine statistics, provides an accurate snapshot of deaths in which the main cause was actually SARS-CoV-2 infection and its short-term consequences (1–3).

### COVID-19 and ethnicity

The main clinical risk factors for COVID-19 mortality are age, male sex, obesity, and some chronic diseases, including in particular renal failure, diabetes, and dementia (4). The effect of ethnicity and migrant status changes according to context (5, 6). Most of the published studies were based on national data or on large cohorts built in the United Kingdom (UK) and the USA. These studies observed an excess of incidence and to a lesser extent of mortality from COVID-19 in the most deprived groups and in some ethnic minorities (7), particularly populations of Asian origin (8). In the UK, the differential was greater for COVID-19 mortality than for other causes of death (5). In Italy, during the first two waves, there was no overall excess of either infection or mortality in the immigrant population (9–12). Both incidence and mortality were lower in immigrants in the first wave of the pandemic. During the second wave, the risk of infection and mortality in immigrants was similar in females and, in males, slightly higher than in Italians. Only for hospitalizations, an excess risk in immigrants was constantly observed (13, 14).

A study conducted in the USA found some differences in the strength of risk factors for infection—but not for severity of disease—in African Americans compared to whites: a higher incidence in children, a greater effect of prior cancer, a lesser effect of crowded housing, and a greater effect of obesity (15).

### Diabetes, obesity, and ethnicity and the effect on COVID-19 mortality

Excess weight and diabetes are negative prognostic factors in COVID-19. A review (16) published at the beginning of the pandemic reported that chronic inflammation, increased coagulation activity, immune response impairment, and potential direct pancreatic damage by SARS-CoV-2 might be among the underlying mechanisms of the association between diabetes and COVID-19 severity. A more recent review (17) focused also on the relationship between body mass index (BMI) and COVID-19-related death and between blood glucose controls on poor COVID-19 outcomes. These factors appear to be useful as a prognostic tool. However, the strength of the association between BMI and COVID-19 severity and mortality varies according to ethnic group (15, 18–20). Diabetes and, in some contexts, obesity show higher prevalence among some ethnic minorities and immigrants from eastern countries, particularly people from South Asia (21). The most deprived populations also often show the poorest glycemic control, possibly because of barriers to effective care and difficulties in reconciling diet regimens and physical activity with economic and time constraints. Nevertheless, epidemiological and clinical evidence suggest that, in South Asians, gaining good glycemic control is more difficult, independently from diet, physical activity, and treatment compliance (22, 23).

Studies carried out in the UK showed that the association between COVID-19 mortality and obesity was stronger in populations originating from South Asia, intermediate in populations of African origin and minor in populations of European origin (19, 24). Differences were reduced by adjusting for pre-clinical conditions (24). This greater effect was also found for people with diabetes. In addition, a study conducted in England in a large cohort of people with diabetes showed that the differences were reduced by adjusting for pre-COVID-19 clinical conditions, suggesting that part of the effect was due to worsened control of diabetes and its complications among the most deprived populations (18). This study also observed that the effect of BMI on COVID-19 mortality was greater in populations of Asian and African origin (18).

Analysis of the effect of BMI and glycemic control in the sub-population of people with diabetes could help to disentangle the associations between ethnicity, diabetes, and COVID-19 mortality. BMI and glycemic control are routinely collected in the Reggio Emilia diabetes registry.

### Objective of the study

The objective of this study was to compare the effect of diabetes and of pathologies potentially related to diabetes on the risk of infection and death from COVID-19 among people from Highly-Developed-Countries (HDCs) including Italians, and immigrants from High-Migratory-Pressure-Countries (HMPCs). Among the population with diabetes, we also aimed to compare the effect of glycemic control and BMI in Italians and immigrants.

## Materials and methods

### Study setting and design

The province of Reggio Emilia has 532,000 inhabitants. A population-based cohort study was conducted. Information was gathered from routine clinical registries and surveillance databases. The study was approved by the Area Vasta Emilia Nord Ethics Committee (Approval No 2020/0045199), which also allowed the population to be included without requiring informed consent, given the retrospective nature of the study.

### Study population

All residents aged over 18 years in the province of Reggio Emilia as of December 31, 2019 were included. The outcomes included all infections reported by COVID-19 surveillance from the beginning of the epidemic (first case diagnosed in Reggio Emilia on February 27, 2020) until August 10, 2021. These cases were then followed up for COVID-19 mortality until September 20, 2021.

### Data sources

Different information sources were used:

- For the resident population, the local health unit resident population registry, updated in real time by the municipal registries from all municipalities within the province; from this source we took information on residence, deaths and country of birth;
- For the outcomes, the local health unit COVID-19 surveillance system, which provided daily data on SARS-CoV-2 infections, hospitalizations and deaths from COVID-19 to the regional coordination structure and from there to the National Institute of Health. Locally, the system was integrated with the laboratory application, the hospitalization management system, the contact tracing management system and mortality registries;
- For the assessment of pre-existing comorbidities, the hospital information system;
- The variables relating to previous cancers were acquired from the cancer registry (25);
- The presence of diabetes, glycated hemoglobin values (HbA1c) and BMI data were acquired from the diabetes registry (26);
- Data about administered vaccines were retrieved from the local health authority vaccine information system.

A detailed description of record linkage operations is described in the report by Mangone and colleagues (25).

## Role of the main variables

### Outcomes

- SARS-CoV-2 infection: positive molecular test (or third-generation antigenic test since December 1, 2020). Only first infections were considered and not re-infections.
- Death from COVID-19: death occurring within 30 days from the diagnosis of positivity to SARS-CoV-2.

We focused on mortality and not on fatality rates to measure the impact of diabetes on COVID-19 severity because, based on the assumption of more limited testing in immigrants and consequent undiagnosed disease, the incidence of infection in immigrants may be underestimated, thus overestimating fatality. Actually, in Emilia-Romagna Region, a reduced probability of testing has been demonstrated, especially for women (9) from HMPC populations (27), and increased screening activity related to international traveling has also been observed in people from HPMCs (28).

## Stratification and exposures

Migrant status was our stratification variable. This was defined, on the basis of country of birth, as high developed countries (HDC) and high migration pressure countries (HMPC). The HDC population included Italians and immigrants from Europe (Andorra, Austria, Belgium, Denmark, Finland, France, Germany, Greece, Ireland, Iceland, Liechtenstein, Luxemburg, Netherlands, Norway, Principality of Monaco, Portugal, Republic of Saint Marin, Spain, Sweden, Switzerland, United Kingdom and Vatican City), North America (United States and Canada), Australia (Australia and New Zeland) and Asia (Israel, Japan and South Korea). Immigrants from all the other countries were considered among the HMPC population (29). Among HMPC a focus was presented about South Asians, because of the known high prevalence of diabetes in this population (21).

A sensitivity analysis restricted to people <65 years of age was performed. The rationale underlying the stratified analysis by age was met in the different age structure of the two populations: people from HMPC had a very low proportion of people over 65. Moreover, a strong interaction was observed between the effect of comorbidity and age on COVID-19 prognosis, thus adjusting may not be sufficient to take into account the difference in age structure.

Type-2 diabetes was our main exposure analysis. In the descriptive analyses, we also considered other diabetes types (Type-1, other, and undefined), but people with other forms of diabetes were excluded from association analyses.

In the sub-population with diabetes, the main determinants of outcomes were the most recent glycemic control and BMI measurements in the period between January 1, 2018, and December 31, 2019. BMI was divided according to the quartiles (<25.90, 25.90–29.07, 29.07–32.90, >32.90).

We also included information about recent comorbidities—such as ischemic heart disease, chronic renal failure, hypertension, obesity, heart failure, arrhythmia, vascular disease and stroke—collected from hospital discharge databases (2015–2019). The Charlson Comorbidity Index (CCI) was calculated based on hospital admissions in the previous 5 years and it was used to evaluate the impact of comorbidity on selected outomes (30). Our CCI incorporated 17 different comorbidities, each of which was weighted according to its potential impact on mortality. This index has been previously applied to COVID-19 patients (31).

Furthermore, the pandemic had different characteristics in different periods: different control measures, different variants, and finally different susceptibility of the populations after the vaccination campaign. For this reason, we added a stratified analysis by calendar period, which may suggest differences if control measures and variants were effect modifiers of the observed associations.

## Adjustment variables

Age, sex and COVID-19 vaccination until August 10, 2021 were used as adjustment variables in the models.

### Statistical analysis

All the analyses were stratified by place of birth, HDC (including Italy), and HMPC. A focus on South Asians known to have the highest risk and prevalence of diabetes was added. Crude COVID-19 incidence and mortality rates (IRs and MRs) were reported. We reported IRs and MRs per 1,000 persons, incidence and mortality in people from HDC and from HMPC were compared through incidence (IRR) and mortality rate ratios (MRR), with associated 95% confidence intervals (95%CI). Hazard ratios (HRs) with their associated 95% CI adjusted for sex and age were calculated using the Cox proportional hazards model. For the outcome of COVID-19 infections only, a further adjustment for COVID-19 vaccination was considered. COVID-19 vaccination was the only variable included as a time-varying covariate in the model. For COVID-19 mortality, this adjustment was not possible because of the limited number of deaths among vaccinated individuals (Table 1), and thus we presented analyses restricted to non-vaccinated individuals instead of adjusting for vaccine status. Analyses restricted to the diabetic population, stratified by country of birth, were also reported.

**Table 1:** Resident population in the province of Reggio Emilia, number of first SARS-CoV-2 infections and deaths from COVID-19, by demographic and clinical characteristics.

**Covariates**			**HDC**					**HMPC**					**South Asian**				
			**Residents**	**SARS-CoV-2infection**		**COVID-19 mortality**		**Residents**	**SARS-CoV-2infection**		**COVID-19 mortality**		**Residents**	**SARS-CoV-2infection**		**COVID-19 mortality**	
			**N**	**N**	**Crude rate** ^*^ **1,000 residents**	**N**	**Crude rate** ^*^ **1,000 residents**	**N**	**N**	**Crude rate** ^*^ **1,000 residents**	**N**	**Crude rate** ^*^ **1,000 residents**	**N**	**N**	**Crude rate** ^*^ **1,000 residents**	**N**	**Crude rate** ^*^ **1,000 residents**
**Overall**			**376,016**	**31,943**	**84.95**	**1,194**	**3.18**	**71,494**	**5,441**	**76.10**	**28**	**0.39**	**12,805**	**1,089**	**85.04**	**8**	**0.62**
Mean age (SD)			53.6 (19.1)	51.3 (19.5)		82.7 (10.0)		43.5 (14.0)	42.8 (13.5)		66.5 (12.2)		40.5 (0.1)	40.2 (0.4)		59.3 (3.8)	
Females			191,222	16,136	84.38	547	2.86	37.561	2,860	76.14	9	0.24	5,113	432		3	
COVID-19 vaccination as of 10/08/21	Not vaccinated	N	82,030	11,229	136.89	1,146	0.01	37.416	2,534	67.73	28	0.75	6,195	422	68.12	8	2.15
		PY	721,014.2	31,394.8		1,187.1		149,829.7	5,886.5		32.3		19,123.4	1088.2		5.4	
	1st dose	N	48.299	13,388	277.19	32	0.66	11.257	1,920	170.56	0	0	2,890	488	168.86	0	0
		PY	41,954.7	40.4		1.3		3,344.8	2.9		0		354.8	0.3		0	
	1st and 2nd dose	N	245,687	7,326	29.82	16	0.07	22.821	987	43.25	0	0	3,720	179	48.12	0	0
		PY	52,304.9	54.0		1.3		2,974.7	4.4		0		272.8	0.5		0	
Diabetes	No		347,480	29,492	84.87	859	2.47	67.364	5,067	75.22	16	0.24	11,599	961	82.85	4	0.34
	Type 1		965	84	87.05	2	2.07	158	10	63.29	0	0	19	0	0	0	0
	Type 2		25,844	2,216	85.75	315	12.19	3.829	349	91.15	12	3.13	1,144	123	107.52	4	3.50
	Other		271	18	66.42	1	3.69	28	2	71.43	0	0	9	1	111.11	0	0
	Undefined		1,456	133	91.35	17	11.68	115	13	113.04	0	0	34	4	117.65	0	0
Ischemic heart disease			8,345	744	89.16	141	16.90	428	37	86.45	2	4.67	110	12	109.09	0	0
Chronic renal failure			2,500	248	99.2	86	34.40	179	21	117.32	0	0	45	5	111.11	0	0
Hypertension			17,231	1,562	90.65	317	18.40	853	90	105.51	4	4.69	155	16	103.23	1	6.45
Obesity			2,385	270	113.21	28	11.74	194	22	113.4	2	10.31	30	5	166.67	1	33.33
Heart failure			5,592	580	103.72	167	29.86	191	17	89.01	1	5.24	39	4	102.56	0	0
Arrhythmia			8,235	783	95.08	180	21.86	259	20	77.22	3	11.58	31	1	32.26	0	0
Vascular diseases			3,015	261	86.57	62	20.56	129	10	77.52	1	7.75	20	3	150.00	0	0
Stroke			7,051	738	104.67	173	24.54	344	41	119.19	1	2.91	69	9	130.43	0	0
Charlson Comorbidity Index	0		344,737	29,151	84.56	669	1.94	69.414	5,256	75.72	22	0.32	12,460	1,046	83.95	7	0.56
	1		13,313	1,217	91.41	222	16.68	1.014	87	85.8	2	1.97	207	26	125.60	0	0
	2		12,494	1,037	83	166	13.29	684	65	95.03	2	2.92	90	9	100.00	0	0
	3		5,472	538	98.32	137	25.04	382	33	86.39	2	5.24	48	8	166.67	1	20.83

February 2020–August 2021.

We decided not to use the term “significant” or “not-significant” since we did not set a significance threshold and we did not performe formal tests of hypothesis. Confidence intervals should be interpreted as continuous variables and should not be used to reject or accept the null hypothesis according to a pre-fixed threshold.

Sensitivity analyses were performed and were restricted to the population <65 years of age.

We added a stratified analysis according to the following four periods of time (1: February 22, 2020–June 1, 2020; 2: June 2, 2020–December 31, 2020; 3: January 1, 2021–June 30, 2021; 4: July 1, 2021–August 10, 2021), depending on the waves and variants of the infection, and these results are shown in the Supplementary material.

**Figure 1:**
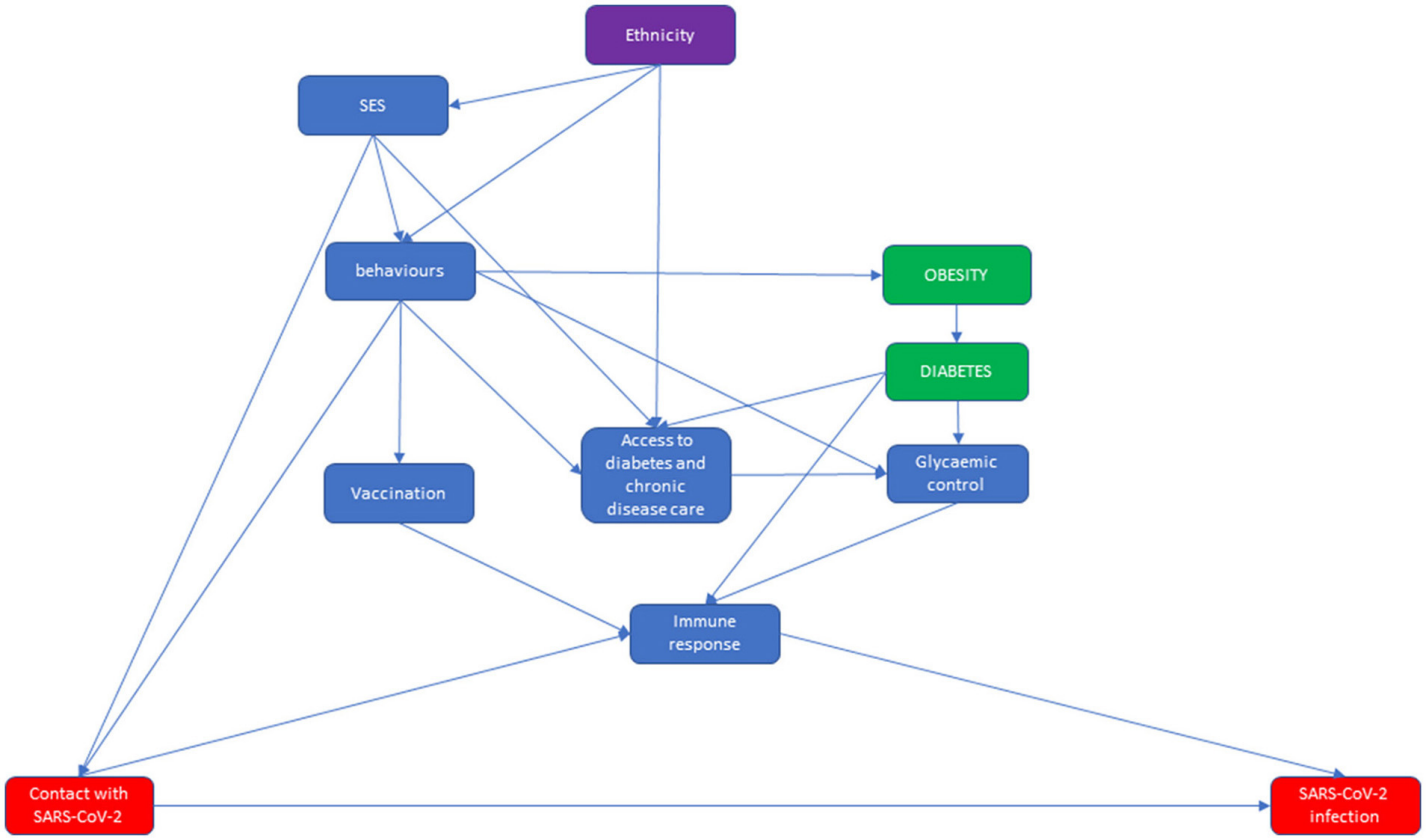
Direct acyclic graph (DAG) of the relationship between ethnicity and diabetes from contact with SARS-CoV-2 to SARS-CoV-2 infection, in which the effect modifier is ethnicity, the exposures are obesity and diabetes, and the outcome is SARS-CoV-2 infection.

**Figure 2:**
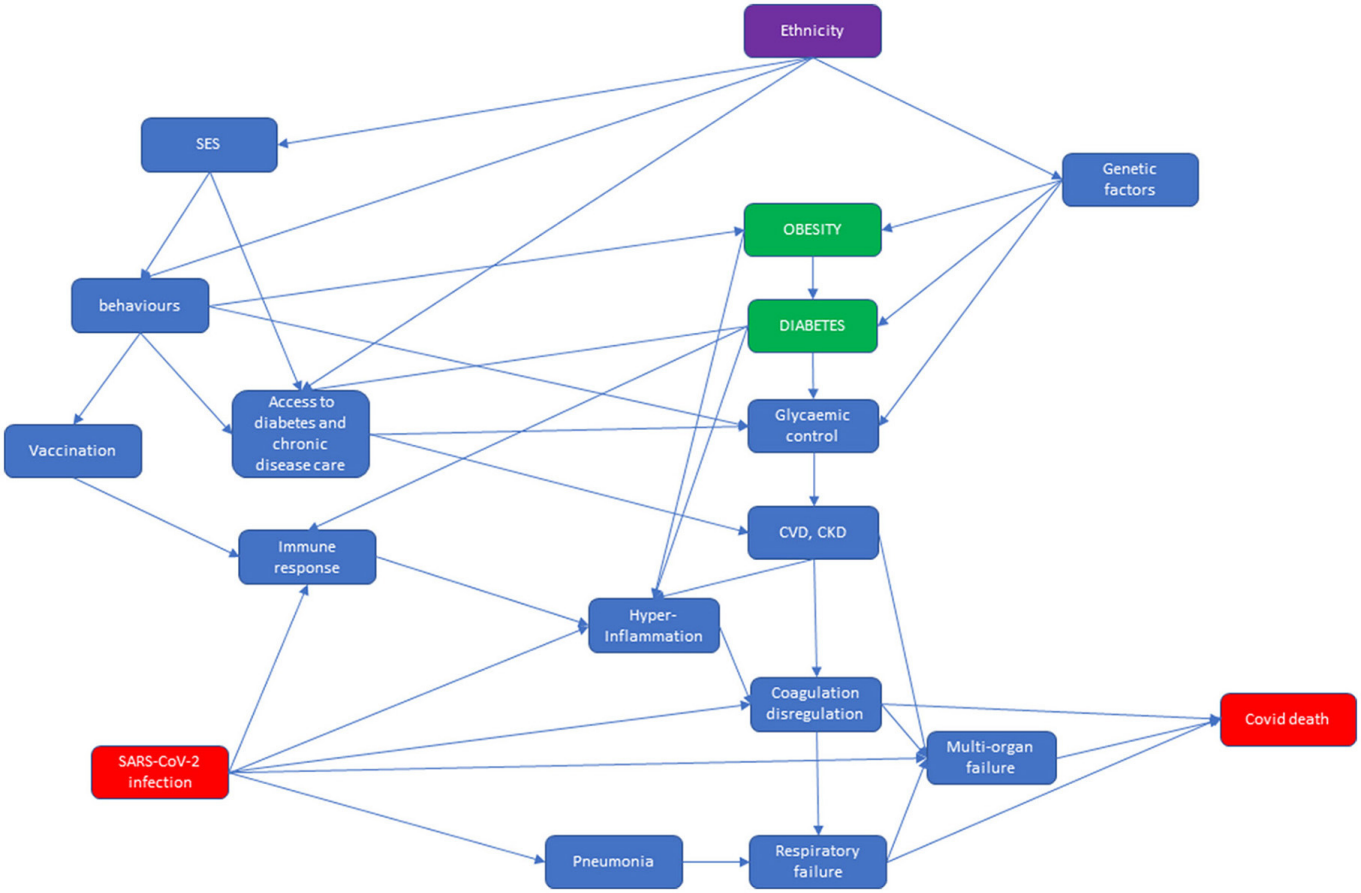
COVID-19 vaccination coverage for HDC and HMPC populations for the adult population residing in Reggio Emilia, as of August 10, 2021.

Two direct acyclic graphs (DAG) were built: the first described the causal network from contact with the virus to infection and the second described the causal network leading from infection to COVID-19-related death (Figures 1, 2).

The first DAG starts from the association between ethnicity and socio-economic status (SES), characterizing immigrant status in Italy for the HMPC population (32). Both ethnicity and SES influence behaviors that can impact obesity [through diet and physical activity (33, 34)], vaccination (35, 36) and—among individuals with diabetes—glycemic control (21) and access to health care (37). Furthermore, linguistic and logistical barriers limit the accessibility of health care for immigrants (37, 38). Behaviors and living and working conditions influence the probability that an individual will be in contact with SARS-CoV-2 (5, 6, 39). Vaccination status, and possibly diabetes (40) and glycemic control (41), influences the ability of the immune system to block the development of a detectable infection once contact has occurred. In this latter step, we had not considered those infections that had not been detected, mostly because they were asymptomatic and did not lead to testing. More generally, the DAG does not account for factors influencing the probability of testing for screening, tracing or mild symptoms. As reported in the rationale for choosing the study outcomes, it is reasonable to think that migrant status could influence the probability of testing (9, 27, 42).

At the top of the second DAG, we have represented pre-existing conditions that might modify the probability of death from COVID-19–reported at the bottom right of the DAG. In Italy, ethnicity is associated with SES, because immigrants have lower SES, which in turn affects behaviors such as physical activity and diet, and they are thus prone to obesity and poor glycemic control. People's behaviors determine vaccination status, which modifies immune response. Moreover, SES and people's behaviors affect access to care for diabetes and other chronic diseases. This is then associated with poor glycemic control and with pre-existing cardiovascular disease (CVD) (37, 43). In addition, genetic factors are linked to diabetes and glycemic control, in particular among the South Asian population (22, 23). Diabetes not only affects the heart and kidneys but hyperglycemia in diabetes is also thought to cause an immune response dysfunction (44). Both these factors are associated with hyperinflammation, coagulation dysregulation, and multi-organ failure (44). SARS-CoV-2 infection is directly associated with pneumonia and respiratory failure, but also with multi-organ failure, and it is indirectly associated with immune response dysfunction, which can evolve into hyperinflammation, and then into coagulation dysregulation. In conclusion, respiratory failure, multi-organ failure, and coagulation dysregulation lead to COVID-19 death (45).

## Results

### Overall population

On December 31, 2019, 447,510 adults (18+) were residents in the province of Reggio Emilia. Of those individuals, 376,016 (84%) were from HDC populations and 71,494 (16%) were from HMPC populations. Generally, the HMPC group was younger than the HDC group, both in terms of the general population and for those with COVID-19. We counted 31,943 COVID-19 infections among HDC and 5,441 among HMPC population. Of these, 1,194 HDC and 28 HMPC died of COVID-19. Table 1 shows crude COVID-19 IRs and MRs per 1,000 individuals in the HDC and HMPC groups according to demographic and clinical characteristics. A lower incidence (IRR: 0.84 95% CI: 0.82–0.87) was observed in the HMPC group than in the HDC group (Supplementary Table 1). The MMR was also lower for HMPC, but it was based on 28 deaths in HMPC and the estimate was rather imprecise (MRR: 0.67 95% CI: 0.46–0.99).

The risk factor that showed a substantial difference in the strength of the association was obesity, which had a greater effect on mortality among HMPC individuals than among the HDC population (HR for HMPC: 18.92 95% CI: 4.48–78.87; HR for HDC: 3.91 95% CI: 2.69–5.69). A slightly higher effect of type-2 diabetes on SARS-CoV-2 infection was observed in the HMPC group (HR: 1.37 95% CI: 1.22–1.53), and particularly in South Asian individuals (HR: 1.43 95% CI: 1.17–1.75), than in the HDC population (HR: 1.20 95% CI: 1.14–1.25), while a difference in the effect on mortality was observed for type-2 diabetes (HR for HMPC: 3.96 95% CI: 1.82–8.60; HR for HDC: 1.71 95% CI: 1.50–1.95) and arrhythmia (HR for HMPC: 5.59 95% CI: 1.61–19.45; HR for HDC: 1.73 95% CI: 1.47–2.04) among the HMPC population compared to the HDC population. Hypertension and vascular diseases also showed a greater impact on risk of death among HMPC individuals than among the HDC population, but the differences were compatible with random fluctuations (Table 2). COVID-19 vaccination was shown to be a protective factor against SARS-CoV-2 infection among both HDC and HMPC populations, with an increasingly protective effect as the number of doses increased (Table 2).

**Table 2:** Hazard ratios (HRs) of first infections from SARS-CoV-2 and deaths from COVID-19, by demographic and clinical characteristics, stratified by HDC, HMPC and South Asian populations.

**Covariates**		**HDC**				**HMPC**				**South Asians (*****N*** = **12,805)**			
		**SARS-CoV-2 infection**		**Death from COVID-19** ^$^		**SARS-CoV-2 infection**		**Death from COVID-19** ^$^		**SARS-CoV-2 infection (*****N*** = **1,089)**		**Death from COVID-19 (N** = **8)**^$^	
		**HR**	**95% CI**	**HR**	**95% CI**	**HR**	**95% CI**	**HR**	**95% CI**	**HR**	**95% CI**	**HR**	**95% CI**
COVID-19 vaccination as of August 10, 2021	Not vaccinated	1		/		1		/		1		/	
	1st dose	0.59	0.54–0.64			0.66	0.49–0.89			0.60	0.26–1.39		
	1st and 2nd dose	0.20	0.18–0.22			0.25	0.16–0.38			0.47	0.17–1.30		
Type-2 diabetes^*^		1.20	1.14–1.25	1.71	1.50–1.95	1.37	1.22–1.53	3.96	1.82–8.60	1.43	1.17–1.75	4.00	0.93–17.20
Ischemic heart disease		1.24	1.15–1.33	1.75	1.46–2.09	1.32	0.95–1.82	2.12	0.49–9.18	1.40	0.78–2.49	/	
Chronic renal failure		1.41	1.24–1.60	2.34	1.87–2.94	1.80	1.15–2.72	/		1.46	0.60–3.52	/	
Hypertension		1.29	1.23–1.36	1.96	1.72–2.24	1.59	1.28–1.96	3.05	1.03–9.06	1.31	0.79–2.16	3.39	0.39–29.18
Obesity		1.43	1.26–1.61	3.91	2.69–5.69	1.61	1.06–2.44	18.92	4.48–79.87	2.14	0.89–5.16	21.91	2.57–184.79
Heart failure		1.51	1.39–1.64	2.01	1.70–2.38	1.34	0.83–2.17	2.00	0.26–15.11	1.30	0.49–3.49	/	
Arrhythmia		1.36	1.27–1.47	1.73	1.47–2.04	1.13	0.73–1.76	5.59	1.61–19.45	0.39	0.05–2.75	/	
Vascular disease		1.19	1.05–1.34	2.05	1.58–2.65	1.13	0.61–2.11	4.28	0.57–32.13	1.86	0.60–5.79	/	
Stroke		1.46	1.35–1.57	2.08	1.77–2.45	1.69	1.25–2.31	1.48	0.20–11.14	1.60	0.83–3.10	/	
Charlson comorbidity index	0	1		1		1		1		1			
	1	1.29	1.22–1.37	2.12	1.81–2.48	1.24	1.00–1.53	1.73	0.40–7.55	1.62	1.09–2.40	/	
	2	1.14	1.07–1.21	2.29	1.92–2.72	1.37	1.07–1.75	2.75	0.64–11.94	1.25	0.64–2.41	/	
	3	1.39	1.28–1.52	3.25	2.69–3.93	1.26	0.89–1.78	4.39	1.01–19.11	2.26	1.12–4.56	10.57	1.21–92.11

HDC, Highly-Developed-Country population; HMPC, High-Migratory-Pressure-Country population.

^*^Type-1 (1,123), other (299) and undefined diabetes (1,571) are not included in the analyses.

^$^The estimates in the models refer only to non-vaccinated people.

The HRs are adjusted for sex, age and COVID-19 vaccination as of August 10, 2021. Reggio Emilia, February 2020–August 2021.

**Figure 3:**
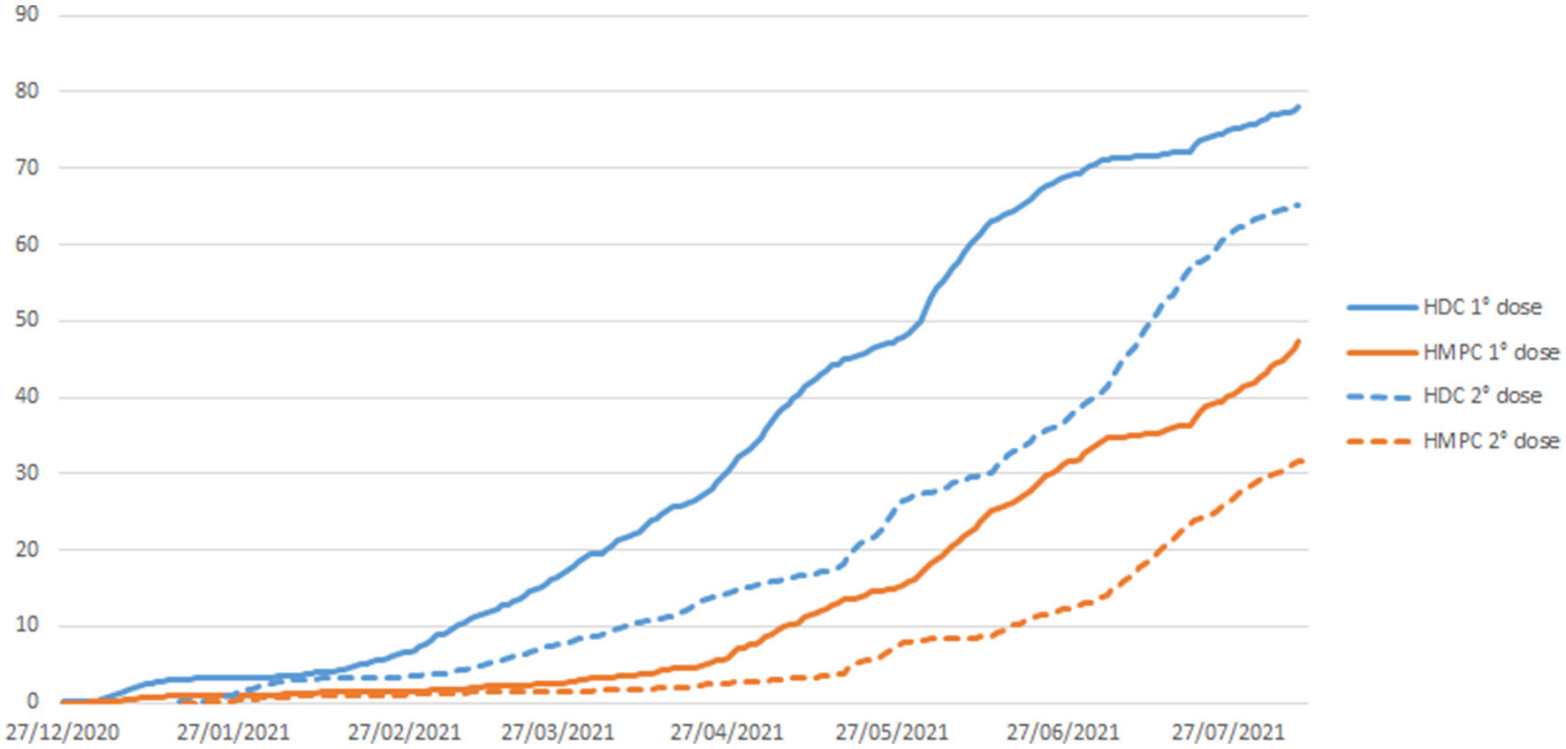
Direct acyclic graph (DAG) of the relationship between ethnicity and diabetes and COVID-19-related death, in which the effect modifier is ethnicity, the exposures are obesity and diabetes, and the outcome is COVID-19-related death.

If we analyzed the initial months of vaccination against COVID-19 in our cohort, cumulative coverage was higher among HDC individuals than HMPC individuals, for both the first and second doses of the vaccine (Figure 3). As of August 10, 2021, one dose vaccination coverage was 78 and 47% for HDC and HMPC, respectively; while the two-dose vaccination coverage was 65 and 31%, respectively. The risk reduction in relation to SARS-CoV-2 infection was around 80% among HDC individuals and 75% among HMPC individuals with two doses of vaccination.

### Diabetic population

Among the residents of the province of Reggio Emilia, 25,844 HDC (87.1%) and 3,829 HMPC (12.9%) individuals had type-2 diabetes (Table 3). Of these, there were 2,216 COVID-19 infections among HDC and 349 among HMPC. COVID-19 deaths were 315 among HDC and 12 among HMPC. Furthermore, the HMPC group showed generally similar incidence (IRR: 0.99 95% CI: 0.88–1.12) and mortality (MRR: 0.89 95% CI: 0.49–1.61) to that of HDC individuals (Supplementary Table 1).

**Table 3:** Population with Type-2 diabetes residing in the province of Reggio Emilia, numbers and percentages of first infections of SARS-CoV-2 and deaths from COVID-19, by demographic and clinical characteristics.

		**Population with type-2 diabetes**			**SARS-CoV-2 infection**		**Death from COVID-19**	
**Covariates**		**HDC**		**HMPC**	**HDC**	**HMPC**	**HDC**	**HMPC**
			**N (%)**	**N (%)**	**N (%)**	**N (%)**	**N (%)**	**N (%)**
**Overall**			**25,844**	**3,829**	**2,216**	**349**	**315**	**12**
Mean age (SD)			72.1 (11.8)	57.9 (11.9)	71.6 (13.2)	55.9 (12.0)	82.2 (8.8)	65.1 (14.3)
Females			11,235 (43.5)	1,833 (47.9)	933 (42.1)	163 (47.0)	135 (42.9)	5 (41.7)
COVID-19 vaccination as of August 10, 2021	Not vaccinated	N	3,812 (14.8)	1,408 (36.8)	696 (31.4)	116 (33.2)	300 (95.2)	12 (100.0)
		PY	53,229.2 (82.5)	8,761.9 (91.1)	2.129,8 (99.5)	423.4 (99.8)	313.7 (99.8)	12.5 (100.0)
	1st dose	N	1,407 (5.4)	350 (9.1)	983 (44.4)	153 (43.8)	11 (3.5)	0 (0.0)
		PY	2,843.9 (4.4)	279.2 (2.9)	2.2 (0.1)	0.1 (0.1)	0.32 (0.1)	0 (0.0)
	1st and 2nd dose	N	20,625 (79.8)	2,071 (54.1)	537 (24.2)	80 (22.9)	4 (1.3)	0 (0.0)
		PY	8,456.3 (13.1)	580.4 (6.0)	9.0 (0.4)	0.6 (0.1)	0.5 (0.2)	0 (0.0)
Ischemic heart disease			2,401 (9.3)	185 (4.8)	246 (11.1)	20 (5.7)	57 (18.1)	1 (8.33
Chronic renal failure			943 (3.7)	64 (1.7)	113 (5.1)	8 (2.3)	45 (14.3)	0 (0.0)
Hypertension			4,456 (17.2)	275 (7.2)	430 (19.4)	31 (8.9)	104 (33.0)	1 (8.3)
Obesity			708 (2.7)	50 (1.3)	100 (4.5)	6 (1.7)	21 (6.7)	1 (8.3)
Heart failure			1,746 (6.8)	71 (1.9)	196 (8.8)	7 (2.0)	62 (19.7)	0 (0.0)
Arrhythmia			1,896 (7.3)	51 (1.3)	213 (9.6)	3 (0.9)	61 (19.4)	1 (8.3)
Vascular disease			798 (3.1)	36 (0.9)	78 (3.5)	2 (0.6)	20 (6.4)	0 (0.0)
Stroke			1,727 (6.7)	90 (2.4)	184 (8.3)	10 (2.9)	51 (16.2)	0 (0.0)
Charlson comorbidity index	0		19,395 (75.1)	3,382 (88.3)	1,580 (71.3)	305 (87.4)	151 (47.9)	10 (83.3)
	1		2,892 (11.2)	227 (5.9)	280 (12.6)	17 (4.9)	58 (18.4)	0 (0.0)
	2		2,150 (8.3)	135 (3.5)	211 (9.5)	17 (4.9)	58 (18.4)	1 (8.3)
	3		1,407 (5.4)	85 (2.2)	145 (6.5)	10 (2.9)	48 (15.24)	1 (8.3)
BMI	<25.90		3,946 (23.1)	748 (29.2)	280 (19.9)	71 (28.2)	39 (24.1)	1 (20.0)
	25.90–29.07		4,246 (24.9)	690 (26.9)	322 (22.9)	76 (30.2)	44 (27.2)	3 (60.0)
	29.07–32.90		4,426 (25.9)	596 (23.2)	371 (26.4)	50 (19.8)	34 (21.0)	0 (0.0)
	>32.90		4,452 (26.1)	531 (20.7)	431 (30.7)	55 (21.8)	45 (27.8)	1 (20.0)
Glycated hemoglobin	≤ 7		13,548 (52.4)	1,409 (36.8)	1,126 (50.8)	142 (40.7)	157 (49.8)	5 (41.7)
	7-8		5,969 (23.1)	816 (21.3)	516 (23.3)	74 (21.2)	76 (24.1)	2 (16.7)
	>8		6,327 (24.5)	1,604 (41,9)	574 (25.9)	133 (38.1)	82 (26.0)	5 (41.67)

HDC, Highly-Developed-Country population; HMPC, High-Migratory-Pressure-Country population.

February 2020–August 2021.

SARS-CoV-2 infection from COVID-19 showed an association with obesity in both HDC and HMPC individuals with diabetes (HRs: 1.73 95% CI 1.41–2.11 and 1.41 95% CI 0.63–3.17, respectively), even if estimates were very imprecise (Table 4). Finally, in individuals with diabetes, the association between the outcomes and BMI showed a consistent trend only in HDC for incidence, with HR for the upper quartiles of BMI (29.07–32.90) 1.13 (95% CI 0.97–1.32) and for BMI>32.90 HR: 1.29 (95% CI 1.10–1.50). For HMPC, no trend was appreciable. The effect of BMI on mortality was only appreciable for BMI >32.90 with HR: 2.04 (95% CI 1.31–3.19) in HDC, while in HMPC only one death was observed in those with BMI >32.90.

**Table 4:** Hazard ratios (HRs) of first infections of SARS-CoV-2 and deaths from COVID-19, in the population with Type-2 diabetes, by clinical characteristics, stratified by HDC and HMPC populations.

		**HDC**				**HMPC**			
		**SARS-CoV-2 infection**		**Death from COVID-19** ^$^		**SARS-CoV-2 infection**		**Death from COVID-19** ^$^	
**Covariates**		**HR**	**95% CI**	**HR**	**95% CI**	**HR**	**95% CI**	**HR**	**95% CI**
COVID-19 vaccination as of August 10, 2021	Not vaccinated	1		/		1		/	
	1st dose	0.96	0.72–1.27			0.54	0.16–1.77		
	1st and 2nd dose	0.40	0.27–0.61			0.55	0.17–1.76		
Obesity		1.73	1.41–2.11	4.36	2.79–6.82	1.41	0.63–3.17	6.43	0.83–49.90
BMI	<25.90	1		1		1		1	
	25.90–29.07	1.04	0.89–1.22	1.12	0.72–1.75	1.14	0.83–1.58	3.30	0.34–31.80
	29.07–32.90	1.13	0.97–1.32	1.09	0.68–1.74	0.87	0.61–1.25	–	
	>32.90	1.29	1.10–1.50	2.04	1.31–3.19	1.12	0.78–1.61	1.50	0.09–25.48
Glycated hemoglobin	≤ 7	1		1		1		1	
	7–8	1.04	0.94–1.16	1.13	0.85–1.49	0.91	0.68–1.20	0.70	0.14–3.60
	>8	1.09	0.98–1.20	1.20	0.91–1.56	0.81	0.64–1.03	0.89	0.26–3.06

HDC, Highly-Developed-Country population; HMPC, High-Migratory-Pressure-Country population.

^$^The estimates in the models refer only to non-vaccinated people.

HRs are adjusted for sex, age and COVID-19 vaccination as of August 10, 2021, Reggio Emilia, February 2020-August 2021.

Glycated hemoglobin showed no association with the two outcomes, except for a slight excess risk of infection for HbA1c levels above 8% appreciable only in HDC and possibly due to random fluctuation (Table 4).

When the analysis was restricted to people <65 years of age, for deaths from COVID-19 among the HDC population, the HR for type-2 diabetes was 3.47 (95% CI: 1.81–6.67), and this became almost double among HMPC, with a HR of 6.12 (95% CI: 1.94–19.32) (Supplementary Table 2).

As for the general population, vaccination against COVID-19 infection also played a protective role among diabetics, resulting in a reduction of 60 and 45%, respectively among HDC and HMPC populations following two doses of vaccination.

Analyses stratified according to four time periods were presented in the Supplementary Tables 3–6.

## Discussion

### Main results

In our cohort, we did not observe any increase in incidence and mortality in individuals from HMPC, compared to Italians and people from HDC. Our data are in line with previous literature that observed a larger association between diabetes and obesity and COVID-19 mortality among immigrants, particularly from South Asia than in Europeans (4, 8, 18, 19). The effect of diabetes on the risk of infection was slightly higher in the HMPC and South Asian populations than in the HDC population. No substantial difference in the strength of the association was observed between obesity or other comorbidities and SARS-CoV-2 infection. Among individuals with diabetes, the Reggio Emilia cohort did not show any difference in the strength of risk and prognostic factors between the HMPC population and the HDC population, although the very small number of deaths in the HMPC population did not allow to draw any certain.

### Limitations of the study and comparison with the international literature

It is worth noting that our results did not confirm the excess of SARS-CoV-2 infections and COVID-19 mortality reported by studies conducted in the UK and the USA for populations of Asian and African origin. Our results are consistent with other Italian studies (46, 47), which also reported a lower incidence of COVID-19 among immigrants (11), while COVID-19 mortality depended on the period considered, alternating phases of slightly lower mortality among immigrants and vice versa (12, 28). This occurred despite the fact that vaccine coverage was lower in the HMPC population than in the HDC population. Stratified analyses by calendar period did not suggest differences in the outcomes for diabetes and pathologies potentially related to diabetes between HDC and HMPC, suggesting control measures at the society level and virus variants were not effect modifiers of the observed associations.

The main limitation of this study is that our estimates of associations concerning the mortality outcome in people from HMPC are extremely imprecise, due to the relatively small number of deaths in this population. A second limitation of the study is that it was not possible to consider the socio-economic and housing conditions of the population, as data on these variables were not available for our cohort. Both types of determinants (socio-economic and clinical) could explain some of the differences in the risk of infection between the HDC and HMPC populations when these differences were present. Studies conducted in the UK showed that by adjusting for socio-economic level, as well as for pre-existing clinical conditions, the excesses of incidence and mortality observed in populations of non-European origin decreased or disappeared, suggesting that some of the worst outcomes were due to the greater state of deprivation of these populations and the worse pre-existing clinical conditions (18, 24). Previous studies found that the effect of comorbidities on COVID-19 mortality was much stronger in younger patients (31). The immigrant population is actually much younger than the Italian population, and part of the phenomenon of the higher impact of specific comorbidities on COVID-19 mortality in immigrants could thus be due to their younger age. With reference to the HDC group, if we restricted the analyses to people aged <65 years, the strength of the associations between diabetes and COVID-19 mortality (and between diabetes, BMI, and COVID-19 mortality, for those individuals with diabetes) was greater than at all ages combined.

Regarding the differences observed in the associations for diabetes between HMPC and HDC populations in SARS-CoV-2 infection, our results were unable to distinguish the portion of the effect caused by SES, behavior and access to care from the portion of the effect caused by the different biological factors and genetic background (Figure 1). However, a previous study on this population (48) had shown that despite the fact that South Asians attended diabetes clinics more than Italians and had a similar level to Italians for compliance with guidelines, they had poorer glycemic control, thus suggesting some biological determinants for the differences observed. Nevertheless we did not observed any overall COVID-19 mortality excess in the HMPC population despite a higher prevalence of diabetes and a stronger effect of diabetes on COVID-19 mortality in this population. This suggests that if any fragility factor due to genetic background is present, its effect on COVID-19 severity is small. The few studies that could adjust, at least in part, for socioeconomic conditions, including crowding and housing conditions found that the excess risk was extremely reduced in immigrants. Furthermore, in studies from UK, part of the excess mortality in non-white people with diabetes was due to worsening pre-existing chronic conditions. It should be considered that immigration in Italy is a relatively recent period, and the healthy migrant effect is still appreciable (48). Thus, it is possible that, even among the immigrants with diabetes, the burden of chronic conditions is still low due to the selection of healthier people among those affording the move to another country (49).

A lower incidence of infection in immigrants may be due to more limited testing and consequent undiagnosed disease. A possible bias in testing has been observed in the Emilia-Romagna Region, but it did not always go in the direction of a lower probability of testing in immigrants. During the first wave, a reduced probability of testing has been demonstrated, especially for women (9) from HMPC populations (27). On the contrary, increased screening activity related to international traveling has been observed in immigrants during the summer of 2020 (28). This difference in the probability of COVID-19 diagnoses led us to not consider the fatality rate of COVID-19 as a reliable outcome to compare the interaction between metabolic risk factors and ethnicity or immigrant status. This is why, despite our conceptual framework presenting the causal chains in two steps, i.e., from contact with the virus to infection and from infection to death, we only presented the associations of factors assessed before infection with infection and with death.

When we focused on individuals with diabetes, the excess in COVID-19 mortality linked to obesity and BMI was similar among the HPMC and HDC populations, as was the effect of glycemic control. This suggests that the stronger effect of obesity on COVID-19 mortality observed in the HMPC population compared with the general population was linked to the higher prevalence of diabetes in the HMPC population. This finding is not consistent with those reported by a large population-based study conducted in a cohort of people with diabetes in England, where the effect of BMI >40 on COVID-19 mortality was stronger among all non-white ethnicities than in those classified as white (11). Nevertheless, our estimates were rather imprecise and confidence intervals included the estimates from the English cohort.

## Conclusions

Diabetes and pathologies potentially related to diabetes are a worse risk factor for death from COVID-19 for HMPC individuals and particularly for South Asians than for the HDC population. Although there is evidence that some biological mechanisms contribute to worsening the outcome of COVID-19 in some ethnic groups, the fact that we did not observe an overall excess risk of COVID-19 mortality in immigrants in our cohort (50) suggests that this intrinsic disadvantage is small and does not justify the higher mortality observed in other studies (5, 7, 51), thus redirecting attention to socio-economic and environmental causes.

## Data availability statement

The data analyzed in this study is subject to the following licenses/restrictions: Researchers who would like to access individual data should present their request, together with a study protocol, to the Area Vasta Emilia Nord Ethics Committee for approval (cereggioemilia@ausl.re.it). Requests to access these datasets should be directed to laura.bonvicini@ausl.re.it.

## Reggio Emilia COVID-19 working group

Massimo Costantini, Roberto Grilli, Massimiliano Marino, Giulio Formoso, Debora Formisano, Ivano Venturi, Cinzia Campari, Francesco Gioia, Serena Broccoli, Marta Ottone, Pierpaolo Pattacini, Giulia Besutti, Valentina Iotti, Lucia Spaggiari, Chiara Seidenari, Licia Veronesi, Paola Affanni, Maria Eugenia Colucci, Andrea Nitrosi, Marco Foracchia, Rossana Colla, Marco Massari, Anna Maria Ferrari, Mirco Pinotti, Nicola Facciolongo, Ivana Lattuada, Laura Trabucco, Stefano De Pietri, Giorgio Francesco Danelli, Laura Albertazzi, Enrica Bellesia, Simone Canovi, Mattia Corradini, Tommaso Fasano, Elena Magnani, Annalisa Pilia, Alessandra Polese, Silvia Storchi Incerti, Piera Zaldini, Efrem Bonelli, Bonanno Orsola, Matteo Revelli, Carlo Salvarani, Carmine Pinto, Pamela Mancuso, Francesco Venturelli, Massimo Vicentini, Cinzia Perilli, Elisabetta Larosa, Eufemia Bisaccia, Emanuela Bedeschi, Alessandro Zerbini, and Paolo Giorgi Rossi.

## Author contributions

PG designed the study, planned the data analysis, drafted the outline of the manuscript, and critically reviewed and revised the manuscript. MO and LBa contributed to designing the study, conducted the analyses, and drafted the methods and results of the manuscript. LBo contributed to designing the study, to analyzing the data, and to writing the manuscript. All authors approved the final manuscript as submitted.
