# Supplementary material for: The effect of diabetes on COVID-19 incidence and mortality: Differences between highly-developed-country and high-migratory-pressure-country populations
Source: Front Public Health. 2023 Mar 8;11:969143. doi: 10.3389/fpubh.2023.969143 (PMC10031649; doi:10.3389/fpubh.2023.969143)
Supplement: Supplementary file 1 [file Data_Sheet_1.pdf]

Supplementary tables

Table S1: Incidence rate ratios (IRRs) of early SARS-CoV-2 infections and mortality rate ratios (MRRs) of deaths from COVID-19, adjusted for sex and age.

	SARS-CoV-2 infection		Death for COVID-19	
	IRR	95% CI	MRR	95% CI
	Overall population			
**HDC**	1		1	
**HMPC**	0.84	0.82-0.87	0.67	0.46-0.99
	Diabetic population			
**HDC**	1		1	
**HMPC**	0.99	0.88-1.12	0.89	0-49-1.61

HDC: Highly-Developed-Country population; HMPC: High-Migratory-Pressure-Country population

Table S2: Hazard Ratios (HRs) of first infections from SARS-CoV-2 and deaths from COVID-19, by clinical characteristics, stratified by HDC and HMPC populations. The HRs are adjusted for sex, age and COVID-19 vaccination as of August the 10th, 2021. <65 years of age. Reggio Emilia, February 2020-August 2021.

Covariates	HDC (N=264.926)				HMPC (N=65.991)			
	SARS-CoV-2 infection (N=24.208)		Death for COVID-19 (N=63)		SARS-CoV-2 infection (N=5,132)		Death for COVID-19 (N=13)	
	HR	95% CI	HR	95% CI	HR	95% CI	HR	95% CI
**Type 2 Diabetes**	1.24	1.15-1.34	3.47	1.81-6.67	1.33	1.17-1.51	6.12	1.94-19.32
**Obesity**	1.24	1.06-1.45	9.36	3.39-25.83	1.61	1.04-2.50	\	
**Charlson Comorbidity Index (0)**	1		1		1		1	
**-1**	1.06	0.95-1.18	9.83	4.81-20.08	1.22	0.96-1.55	\	
**-2**	0.99	0.89-1.10	6.52	2.87-14.78	1.32	0.99-1.75	6.48	0.82-51.11
**-3**	1.10	0.92-1.31	24.99	11.88-52.54	1.19	0.79-1.77	8.90	1.12-70.51

HDC: Highly-Developed-Country population; HMPC: High-Migratory-Pressure-Country population

Table S3: Number of first SARS-CoV-2 infections and deaths from COVID-19 by immigrant status and periods, Reggio Emilia.

	Period 1		Period 2		Period 3		Period 4	
	**HDC**	**HMPC**	**HDC**	**HMPC**	**HDC**	**HMPC**	**HDC**	**HMPC**
**SARS-CoV-2 infection**	4,082	427	12,790	2,131	14,188	2,647	883	236
**Death for COVID-19**	536	11	340	6	314	11	4	0

HDC: Highly-Developed-Country population; HMPC: High-Migratory-Pressure-Country population

Table S4: Hazard Ratios (HRs) of first infections from SARS-CoV-2, by demographic and clinical characteristics and periods, stratified for Italian and foreign populations. The HRs are adjusted for sex, age and COVID-19 vaccination as of August the 10th, 2021, Reggio Emilia.

Covariates	Period 1					
	HDC		HMPC		South Asians	
	HR	95% CI	HR	95% CI	HR	95% CI
**Type 2 Diabetes**	1.34	1.22-1.47	2.21	0.55-8.88	/	
**Ischemic heart disease**	1.27	1.09-1.48	2.39	1.12-5.12	4.92	1.07-22.60
**Chronic renal failure**	1.35	1.05-1.73	3.44	1.28-9.24	6.65	0.88-50.30
**Hypertension**	1.61	1.46-1.78	2.06	1.17-3.62	3.70	0.83-16.52
**Obesity**	2.14	1.66-2.75	2.14	0.69-6.68	/	
**Heart failure**	1.91	1.65-2.20	1.35	0.33-5.45	/	
**Arrhythmia**	1.59	1.39-1.82	2.50	1.03-6.10	/	
**Vascular diseases**	1.28	1.01-1.64	2.20	0.55-8.84	/	
**Stroke**	1.46	1.35-1.57	1.69	1.25-2.31	/	
**Charlson Comorbidity Index (0)**	1		1		1	
**-1**	1.69	1.50-1.89	1.96	1.12-3.44	3.16	0.72-13.84
**-2**	1.67	1.48-1.88	2.05	1.09-3.86	/	

**-3**	2.18	1.87-2.54	1.52	0.57-4.09	6.22	0.81-47.93
**Covariates**	**Period 2**					
	**HDC**		**HMPC**		**South Asians**	
	HR	95% CI	HR	95% CI	HR	95% CI
**Type 2 Diabetes**	1.18	1.09-1.27	1.54	1.30-1.83	1.69	0.47-5.48
**Ischemic heart disease**	1.17	1.03-1.32	1.55	0.97-2.48	1.15	0.42-3.11
**Chronic renal failure**	1.40	1.14-1.72	1.40	0.67-2.94	1.45	0.36-5.82
**Hypertension**	1.15	1.05-1.26	1.43	1.01-2.02	1.04	0.43-2.54
**Obesity**	1.38	1.14-1.68	2.03	1.12-3.67	2.15	0.53-8.68
**Heart failure**	1.22	1.05-1.42	1.74	0.90-3.36	1.62	0.40-6.56
**Arrhythmia**	1.19	1.05-1.35	1.13	0.56-2.26	1.03	0.14-7.38
**Vascular diseases**	1.27	1.04-1.54	1.39	0.58-3.35	1.56	0.22-11.10
**Stroke**	1.46	1.35-1.57	1.69	1.25-2.31	/	
**Charlson Comorbidity Index (0)**	1		1		1	
**-1**	1.18	1.07-1.31	1.20	0.86-1.69	1.25	0.62-2.54
**-2**	1.04	0.94-1.16	1.33	0.89-1.97	0.72	0.18-2.92
**-3**	1.22	1.05-1.42	1.43	0.86-2.38	2.13	0.67-6.66
**Covariates**	**Period 3**					
	**HDC**		**HMPC**		**South Asians**	
	HR	95% CI	HR	95% CI	HR	95% CI
**Type 2 Diabetes**	1.09	1.01-1.18	1.32	1.12-1.56	/	
**Ischemic heart disease**	1.25	1.11-1.41	0.76	0.41-1.41	1.28	0.57-2.89
**Chronic renal failure**	1.36	1.11-1.68	1.62	0.84-3.13	1.01	0.25-4.07
**Hypertension**	1.16	1.06-1.27	1.50	1.09-2.06	1.31	0.67-2.56
**Obesity**	1.20	0.98-1.47	1.07	0.51-2.24	1.55	0.39-6.23
**Heart failure**	1.35	1.17-1.55	1.02	0.46-2.28	1.18	0.29-4.75
**Arrhythmia**	1.21	1.07-1.37	0.84	0.40-1.77	/	
**Vascular diseases**	1.01	0.81-1.24	0.72	0.23-2.34	2.24	0.56-8.99
**Stroke**	1.46	1.35-1.57	1.69	1.25-2.31	/	
**Charlson Comorbidity Index (0)**	1		1		1	
**-1**	1.14	1.03-1.26	1.07	0.77-1.50	1.68	1.00-2.83
**-2**	0.97	0.88-1.08	1.31	0.91-1.89	1.69	0.80-3.58
**-3**	1.11	0.96-1.30	1.04	0.60-1.79	2.04	0.76-5.48
**Covariates**	**Period 4**					
	**HDC**		**HMPC**		**South Asians**	
	HR	95% CI	HR	95% CI	HR	95% CI
**Type 2 Diabetes**	0.99	0.62-1.59	1.21	0.55-2.64	0.78	0.09-6.43
**Ischemic heart disease**	0.31	0.08-1.23	3.65	0.89-15.04	/	
**Chronic renal failure**	/		3.83	0.53-27.57	/	
**Hypertension**	1.07	0.61-1.87	3.51	1.28-9.66	/	
**Obesity**	1.85	0.83-4.13	2.91	0.41-20.83	38.44	4.85-304.96
**Heart failure**	0.76	0.24-2.37	/		/	
**Arrhythmia**	1.25	1.28-3.95	/		/	
**Vascular diseases**	0.39	0.05-2.77	/		/	
**Stroke**	1.46	1.35-1.57	1.69	1.25-2.31	/	
**Charlson Comorbidity Index (0)**	1		1		1	
**-1**	0.85	0.45-1.61	2.12	0.78-5.74	4.21	0.55-32.52
**-2**	0.70	0.35-1.41	/		/	
**-3**	0.97	0.40-2.37	1.59	0.22-11.40	/	

HDC: Highly-Developed-Country population; HMPC: High-Migratory-Pressure-Country population

Table S5: Number of first infections of SARS-CoV-2 and deaths from COVID-19 by period in the population with Type-2 diabetes, Reggio Emilia.

	**Period 1**		**Period 2**		**Period 3**		**Period 4**	
	**HDC**	**HMPC**	**HDC**	**HMPC**	**HDC**	**HMPC**	**HDC**	**HMPC**

**SARS-CoV-2 infection**	546	25	837	156	813	161	20	7
**Death for COVID-19**	127	6	101	0	86	6	1	0

HDC: Highly-Developed-Country population; HMPC: High-Migratory-Pressure-Country population

Table S6: Hazard ratios (HRs) of first infections from SARS-CoV-2, in the population with Type-2 diabetes, by clinical characteristics and periods, stratified by HDC and HMPC populations. HRs are adjusted for sex, age and COVID-19 vaccination as of August the 10th, 2021, Reggio Emilia.

Covariates	Period 1				Period 2				Period 3				Period 4			
	HDC		HMPC		HDC		HMPC		HDC		HMPC		HDC		HMPC	
	HR	95% CI	HR	95% CI	HR	95% CI	HR	95% CI	HR	95% CI	HR	95% CI	HR	95% CI	HR	95% CI
**Obesity**	2.69	1.88-3.85	/		1.79	1.30-2.47	1.06	0.26-4.29	1.21	0.83-1.77	1.54	0.49-4.84	/		13.10	1.55-110.63
**BMI (<25.90)**	1		1		1		1		1		1		1		/	
**(25.90-29.07)**	0.91	0.63-1.31	1.53	0.48-4.82	1.03	0.80-1.33	1.16	0.70-1.92	1.16	0.90-1.50	1.05	0.66-1.66	0.37	0.10-1.40	/	
**(29.07-32.90)**	1.01	0.70-1.45	0.26	0.03-2.25	1.17	0.9-1.50	1.15	0.68-1.95	1.22	0.95-1.57	0.74	0.43-1.25	/		/	
**(>32.90)**	1.37	0.97-1.95	0.93	0.21-4.05	1.36	1.07-1.74	1.51	0.89-2.56	1.22	0.95-1.57	0.81	0.47-1.41	0.77	0.25-2.32	/	
**Glycated hemoglobin (<=7)**	1		1		1		1		1		1		1		1	
**(7-8)**	0.96	0.77-1.19	0.53	0.17-1.62	1.07	0.90-1.26	0.89	0.59-1.35	1.06	0.90-1.27	1.01	0.67-1.53	1.23	0.45-3.32	0.87	0.08-9.60
**(>8)**	1.21	0.98-1.47	0.81	0.22-1.30	0.97	0.82-1.14	0.72	0.50-1.03	1.12	0.95-1.32	0.95	0.67-1.35	0.39	0.09-1.79	1.56	0.28-8.78

HDC: Highly-Developed-Country population; HMPC: High-Migratory-Pressure-Country population
